# NIH Launches Genes and Environment Initiative

**Published:** 2006-04

**Authors:** Tanya Tillett

The NIEHS and its fellow NIH institutes and centers have joined forces to reveal still more connections between genes, the environment, and human health. On 6 February 2006 Health and Human Services Secretary Mike Leavitt announced that the Genes and Environment Initiative (GEI) would receive $68 million in fiscal year 2007, a $40 million increase above the funding already planned for these NIH research efforts. The GEI will seek to speed up research to uncover the genetic roots of common human diseases such as asthma, arthritis, and Alzheimer disease.

“This initiative would not have been possible a year or two ago,” said NIH director Elias Zerhouni in an announcement of the initiative’s launch. “This is a tangible result of the nation’s increased investment in medical research over the past ten years. . . . We stand on the threshold of creating a future that will revolutionize the practice of medicine by allowing us to predict disease, develop more precise therapies and, ultimately, preempt the development of disease in the first place.”

The GEI will be managed by a coordinating committee headed by NIEHS director David A. Schwartz and Francis S. Collins, director of the National Human Genome Research Institute.

The proposed federal funding will enable the GEI to perform genotyping studies for several dozen common diseases, which will be selected by peer review. The NIH also expects to invest in and develop four new environmental monitoring tests and devices each year to measure toxicant exposures, dietary intake, and physical activity, and to determine individuals’ biological responses to those influences. Eventually these new tools may be applied to population studies to speed up data processing, enhance data accuracy, and reduce costs. The National Center for Bio-technology Information, a part of the National Library of Medicine, will develop databases to manage the vast amount of genetic, medical, and environmental information that is expected to be generated from the initiative.

At a press conference announcing the GEI, Schwartz said the new monitors will focus on more precise measures of environmental exposure, giving researchers an edge in determining how risk factors interact with specific genotypes to either maintain health or lead to disease. “This is a whole program,” he says. “This is a puzzle [of] understanding the relationship between genetic variation and environmental variation, and the more that we can inform environmental variation as the genetic studies move along, the more we’ll understand why certain individuals develop disease.”

Brenda Weis, senior science advisor at the NIEHS, sees great potential in the new research effort. “It is my belief that the GEI will accelerate the pace of discovery about the role of genes and the environment in the development of human disease,” she says. “The GEI builds on the knowledge gained through the Human Genome Project and the HapMap Project, and will provide, for the first time, personalized measures of exposure with the same level of precision as we have for genomic analyses.”

